# SFPEL-LPI: Sequence-based feature projection ensemble learning for predicting LncRNA-protein interactions

**DOI:** 10.1371/journal.pcbi.1006616

**Published:** 2018-12-11

**Authors:** Wen Zhang, Xiang Yue, Guifeng Tang, Wenjian Wu, Feng Huang, Xining Zhang

**Affiliations:** 1 College of Informatics, Huazhong Agricultural University, Wuhan, China; 2 School of Computer Science, Wuhan University, Wuhan, China; 3 Department of Computer Science and Engineering, The Ohio State University, Columbus, United States of America; 4 Electronic Information School, Wuhan University, Wuhan, China; Ottawa University, CANADA

## Abstract

LncRNA-protein interactions play important roles in post-transcriptional gene regulation, poly-adenylation, splicing and translation. Identification of lncRNA-protein interactions helps to understand lncRNA-related activities. Existing computational methods utilize multiple lncRNA features or multiple protein features to predict lncRNA-protein interactions, but features are not available for all lncRNAs or proteins; most of existing methods are not capable of predicting interacting proteins (or lncRNAs) for new lncRNAs (or proteins), which don’t have known interactions. In this paper, we propose the sequence-based feature projection ensemble learning method, “SFPEL-LPI”, to predict lncRNA-protein interactions. First, SFPEL-LPI extracts lncRNA sequence-based features and protein sequence-based features. Second, SFPEL-LPI calculates multiple lncRNA-lncRNA similarities and protein-protein similarities by using lncRNA sequences, protein sequences and known lncRNA-protein interactions. Then, SFPEL-LPI combines multiple similarities and multiple features with a feature projection ensemble learning frame. In computational experiments, SFPEL-LPI accurately predicts lncRNA-protein associations and outperforms other state-of-the-art methods. More importantly, SFPEL-LPI can be applied to new lncRNAs (or proteins). The case studies demonstrate that our method can find out novel lncRNA-protein interactions, which are confirmed by literature. Finally, we construct a user-friendly web server, available at http://www.bioinfotech.cn/SFPEL-LPI/.

## Author summary

LncRNA-protein interactions play important roles in post-transcriptional gene regulation, poly-adenylation, splicing and translation. Identification of lncRNA-protein interactions helps to understand lncRNA-related activities. In this paper, we propose a novel computational method “SFPEL-LPI” to predict lncRNA-protein interactions. SFPEL-LPI makes use of lncRNA sequences, protein sequences and known lncRNA-protein associations to extract features and calculate similarities for lncRNAs and proteins, and then combines them with a feature projection ensemble learning frame. SFPEL-LPI can predict unobserved interactions between lncRNAs and proteins, and also can make predictions for new lncRNAs (or proteins), which have no interactions with any proteins (or lncRNAs). SFPEL-LPI produces high-accuracy performances on the benchmark dataset when evaluated by five-fold cross validation, and outperforms state-of-the-art methods. The case studies demonstrate that SFPEL-LPI can find out novel associations, which are confirmed by literature. To facilitate the lncRNA-protein interaction prediction, we develop a user-friendly web server, available at http://www.bioinfotech.cn/SFPEL-LPI/.

This is a *PLOS Computational Biology* Methods paper.

## Introduction

Long noncoding RNAs (lncRNAs) are a class of transcribed RNA molecules with a length of more than 200 nucleotides that do not encode proteins [1,2]. Since lncRNAs are involved in important biological regulations [3–5], lncRNAs have gained widespread attention. Studies [5–9] revealed that lncRNAs can interact with proteins, and then activate post-transcriptional gene regulation, poly-adenylation, splicing and translation. Identification of lncRNA-protein interactions helps to understand lncRNAs’ functions. There exist a large number of unexplored lncRNAs and proteins, which makes it impossible to examine their interactions efficiently and effectively through wet experiments.

In recent years, many computational methods have been proposed to predict lncRNA-protein interactions, in order to screen lncRNA-protein interactions and guide wet experiments. There are two types of computational methods: binary classification methods and semi-supervised learning methods. The binary classification methods take known interacting lncRNA-protein pairs as positive instances and non-interacting pairs as negative instances, and build binary classification-based models. Muppirala et al. [10] adopted the k-mer composition to encode RNA sequences and protein sequences, and used SVM and random forest to build prediction models. Wang et al. [11] used RNA-protein interactions as positive instances, and randomly selected twice number of protein-RNA pairs without interaction information as negative samples, and then built prediction models by using naive Bayes. Suresh et al. [12] proposed a support vector machine-based predictor “RPI-Pred” to predict protein-RNA interactions based on their sequences and structures. Xiao et al. [13] used the HeteSim measure to score lncRNA-protein pairs, and then built an SVM classifier based on HeteSim scores. However, binary classification-based methods are influenced by the imbalance ratio between positive instances and negative instances, and how to select high-quality negative instances is challenging. Semi-supervised learning methods formulate the lncRNA-protein interaction prediction as semi-supervised learning tasks. Lu et al. [14] used matrix multiplication to score each RNA-protein pair for prediction. Li et al. [15] proposed a heterogeneous network-based method “LPIHN”, which integrated the lncRNA-lncRNA similarity network, the lncRNA-protein interaction network and the protein-protein interaction network. Then, a random walk with restart was implemented on the heterogeneous network to infer lncRNA-protein interactions. Yang et al. [16] proposed the Hetesim algorithm, which can predict lncRNA-protein relation based on the heterogeneous lncRNA-protein network. Ge et al. [17] proposed a computational method “LPBNI” based on the lncRNA-protein bipartite network inference. Zheng et al. [18] constructed multiple protein-protein similarity networks to predict lncRNA-protein interactions. Zhang et al. [19] employed KATZ measure to calculate similarities between lncRNAs and proteins in a global network, which were constructed based on lncRNA-lncRNA similarity, lncRNA-protein associations and protein-protein interactions. Hu et al. [20] presented the eigenvalue transformation-based semi-supervised link prediction method “LPI-ETSLP”. Zhang et al. [21] proposed a linear neighborhood propagation method (LPLNP) by combining interaction profiles, expression profiles, sequence composition of lncRNAs and interaction profile, CTD feature of proteins. Moreover, there are related works about the DNA-protein binding prediction [22,23].

Existing computational methods utilize diverse lncRNA features and protein features, but features are not available for all lncRNAs or proteins, and these methods cannot work when information is unavailable. In addition, many lncRNAs (or proteins) don’t have known interactions with any protein (or lncRNA), and we name them as new lncRNAs (or proteins). Most existing methods are not capable of predicting interacting proteins (or lncRNAs) for new lncRNAs (or proteins).

In this paper, we propose the sequence-based feature projection ensemble learning method, “SFPEL-LPI”, to predict lncRNA-protein interactions. First, SFPEL-LPI extracts lncRNA sequence-based features and protein sequence-based features. Second, SFPEL-LPI calculates multiple lncRNA-lncRNA similarities and protein-protein similarities by using lncRNA sequences, protein sequences and known lncRNA-protein interactions. Then, SFPEL-LPI combines multiple similarities and multiple features with a feature projection ensemble learning frame. Computational experiments demonstrate that SFPEL-LPI predicts lncRNA-protein associations accurately and outperforms other state-of-the-art methods. More importantly, SFPEL-LPI can be applied to new lncRNAs (or proteins). The case studies demonstrate that our method can find out novel lncRNA-protein interactions.

## Materials and methods

### Dataset

Several databases facilitate the lncRNA-protein interaction prediction. NPInter database [24] includes experimental interactions among non-coding RNA and biomolecules (i.e. proteins, genomic DNAs and RNAs). NONCODE is an integrated information resource for non-coding RNAs. SUPERFAMILY [25] is a database of structural and functional annotation for all proteins and genomes. As far as we know, lncRNA-protein interactions from NPInter v2.0 database were widely used in related studies [20,21,26–29]. Based on NPInter v2.0 interactions, we compiled a dataset containing 4158 lncRNA-protein interactions between 990 lncRNAs and 27 proteins. Moreover, we collected the sequences of these lncRNAs and proteins from NONCODE and SUPERFAMILY respectively. We adopt NPInter v2.0 dataset as the benchmark dataset to test the performances of prediction models.

Here, we introduce notations about the dataset. Given a set of lncRNAs $L=\{L_{1},L_{2},\cdots ,L_{s}\}$ and a set of proteins $P=\{P_{1},P_{2},\cdots ,P_{t}\}$, known lncRNA-protein interactions can be represented by an *s*×*t* interaction matrix *Y*, where *Y*_*ij*_ = 1 if the lncRNA *L*_*i*_ interacts with the protein *P*_*j*_, otherwise *Y*_*ij*_ = 1.

### Features for lncRNAs and proteins

In this section, we describe two lncRNA features and two protein features, based on lncRNA sequences, protein sequences and known lncRNA-protein interactions. On one hand, a great number of features [30–36] can be extracted from lncRNAs sequences and proteins sequences, and feature-extraction tools such as Pse-in-One[37], BioSeq-Analysis[38], repRNA[39] [40], iMiRNA-PseDPC [41] and UltraPse [42] have been available. One the other hand, known lncRNA-protein interactions can bring features to describe lncRNAs and proteins.

#### LncRNA features

The pseudo dinucleotide composition (PseDNC) [43–46] describes the contiguous local sequence-order information and the global sequence-order information of lncRNAs. The pseudo dinucleotide composition has several variants, and we use the parallel correlation pseudo dinucleotide composition, which contains the occurrences of different dinucleotides and the physicochemical properties of dinucleotides. The PseDNC feature vector of an RNA sequence *L* is defined as:

$$L=[d_{1},d_{2},\cdots ,d_{16},{d_{16+1},\cdots ,d}_{16+\tau }]$$

where

$$d_{k}=\left\{ \begin{array}{c} \frac{f_{k}}{\sum_{i=1}^{16}f_{i}+w\sum_{j=1}^{\tau }\theta_{j}}1\leq k\leq 16\\ \frac{w\theta_{k-16}}{\sum_{i=1}^{16}f_{i}+w\sum_{j=1}^{\tau }\theta_{j}}17\leq k\leq 16+\tau \end{array}\right.$$

where *f*_*k*_ is the normalized occurrence frequency of dinucleotide in the RNA sequence *L*; the parameter *τ* is an integer, representing the highest counted rank of the correlation along an RNA sequence; *w* is the weight factor ranging from 0 to 1; *θ*_*j*_ is the *j*-tier correlation factor reflecting the sequence-order correlation between all the *j*-th most contiguous dinucleotides along an RNA sequence. We obtain PseDNC feature vectors of lncRNAs by using the python package "repDNA”, and more details about PseDNC are described in [40].

Moreover, we define the interaction profiles (IP) of lncRNAs based on known lncRNA-protein interactions. For a lncRNA *L*_*i*_, its interaction profile is a binary vector encoding the presence or absence of interactions with every protein, denoted as ${IP}_{L_{i}}$. Actually, the interaction profile of a lncRNA corresponds to a row vector of the interaction matrix *Y*, ${IP}_{L_{i}}=Y(i,:)$.

#### Protein features

The pseudo amino acid composition (PseAAC) [47–49] describes the amino acid composition and the sequence-order information of proteins, and has been widely used for tasks in bioinformatics. PseAAC contains 20 components reflecting the occurrence frequency of amino acids in a protein as well as the additional factors reflecting sequence-order information. Thus, we use PseAAC as a feature to represent proteins. There are several variants of PseAAC, and we adopt the parallel correlation pseudo amino acid composition. The PseAAC feature vector of a protein sequence *P* is defined as:

$$P=[x_{1},x_{2},\cdots ,x_{20},x_{20+1},\cdots ,x_{20+\tau }]$$

where

$$x_{u}=\left\{ \begin{array}{c} \frac{f_{u}}{\sum_{i=1}^{20}f_{i}+w\sum_{j=1}^{\tau }\theta_{j}}1\leq u\leq 20\\ \frac{w\theta_{u-20}}{\sum_{i=1}^{20}f_{i}+w\sum_{j=1}^{\tau }\theta_{j}}21\leq u\leq 20+\tau \end{array}\right.$$

where *f*_*i*_ is the normalized occurrence frequency of the 20 amino acids in the protein sequence *P*; the parameter *τ* is an integer, representing the highest counted rank of the correlation along a protein sequence; *w* is the weight factor ranging from 0 to 1; *θ*_*j*_ is the *j*-tier correlation factor reflecting the sequence-order correlation between all the *j*-th most contiguous residues along a protein sequence. We obtain the PseAAC feature vectors of proteins by using web server “Pse-in-One”, and more details are described in [37].

Similar to the lncRNA interaction profiles, the protein interaction profile (IP) of a protein *P*_*i*_ is a binary vector specifying the presence or absence of interactions with every lncRNAs, denoted as ${IP}_{p_{i}}$. The interaction profile of a protein corresponds to a column vector of the interaction matrix *Y*, ${IP}_{p_{i}}=Y(:,i)$.

### Similarities for lncRNAs and proteins

In this section, we describe three lncRNA-lncRNA similarities and three protein-protein similarities.

#### LncRNA-lncRNA similarities

As introduced in Section “LncRNA features”, we have two lncRNA features: PseDNC and IP, and thus use them to calculate two types of lncRNA-lncRNA similarities. There are different approaches to calculate similarity based on feature vectors, such as Jaccard similarity, Gauss similarity and cosine similarity. Here, we adopt the linear neighborhood similarity (LNS), which has been proposed in our previous work and successfully applied to many bioinformatics problems [21,34,50].

Moreover, we define the Smith Waterman subgraph similarity (SWSS) for lncRNAs. Smith Waterman algorithm [51] is a powerful tool to calculate similarity between biological sequences, but Smith Waterman algorithm only takes the sequence information into account. By considering sequence information and interactions information, we define Smith Waterman subgraph similarity (SWSS) between lncRNA *L*_*i*_ and lncRNA *L*_*j*_ as,

$$SWSS(L_{i},L_{j})=\sum_{P_{o1}\in A(L_{i})}\sum_{P_{o2}\in A(L_{j})}\frac{SW(P_{o1},P_{o2})}{n1\times n2}$$ (15

where *SW*(*P*_*o*1_,*P*_*o*2_) is the Smith Waterman score between protein *P*_*o*1_ and protein *P*_*o*2_. A(*L*_*i*_) and A(*L*_*j*_) are the set of proteins which interact with *L*_*i*_ and *L*_*j*_. *n*1 = |A(*L*_*i*_)| and *n*2 = |A(*L*_*j*_)|.

Therefore, we obtain three lncRNA-lncRNA similarities: PseDNC similarity, IP similarity and SWSS similarity.

#### Protein-protein similarities

As introduced in Section “Protein features”, we have two proteins features: PseAAC and IP. We also calculate two types of similarities by using the linear neighborhood similarity measure.

Similarly, we can calculate the Smith Waterman Subgraph Similarity (SWSS) between two proteins *P*_*i*_ and *P*_*j*_,

$$SWSS(P_{i},P_{j})=\sum_{L_{o1}\in A(P_{i})}\sum_{L_{o2}\in A(P_{j})}\frac{SW(L_{o1},L_{o2})}{m1\times m2}$$ (2)

where *SW*(*P*_*o*1_,*P*_*o*2_) is the Smith Waterman score between lncRNA *L*_*o*1_ and lncRNA *L*_*o*2_. A(*P*_*i*_) and A(*P*_*j*_) are the set of lncRNAs which interact with protein *P*_*i*_ and protein *P*_*j*_. *m*1 = |A(*P*_*i*_)| and *m*2 = |A(*P*_*j*_)|.

Therefore, we obtain three protein-protein similarities: PseAAC similarity, IP similarity and SWSS similarity.

### Feature projection ensemble learning method

**Fig 1:**
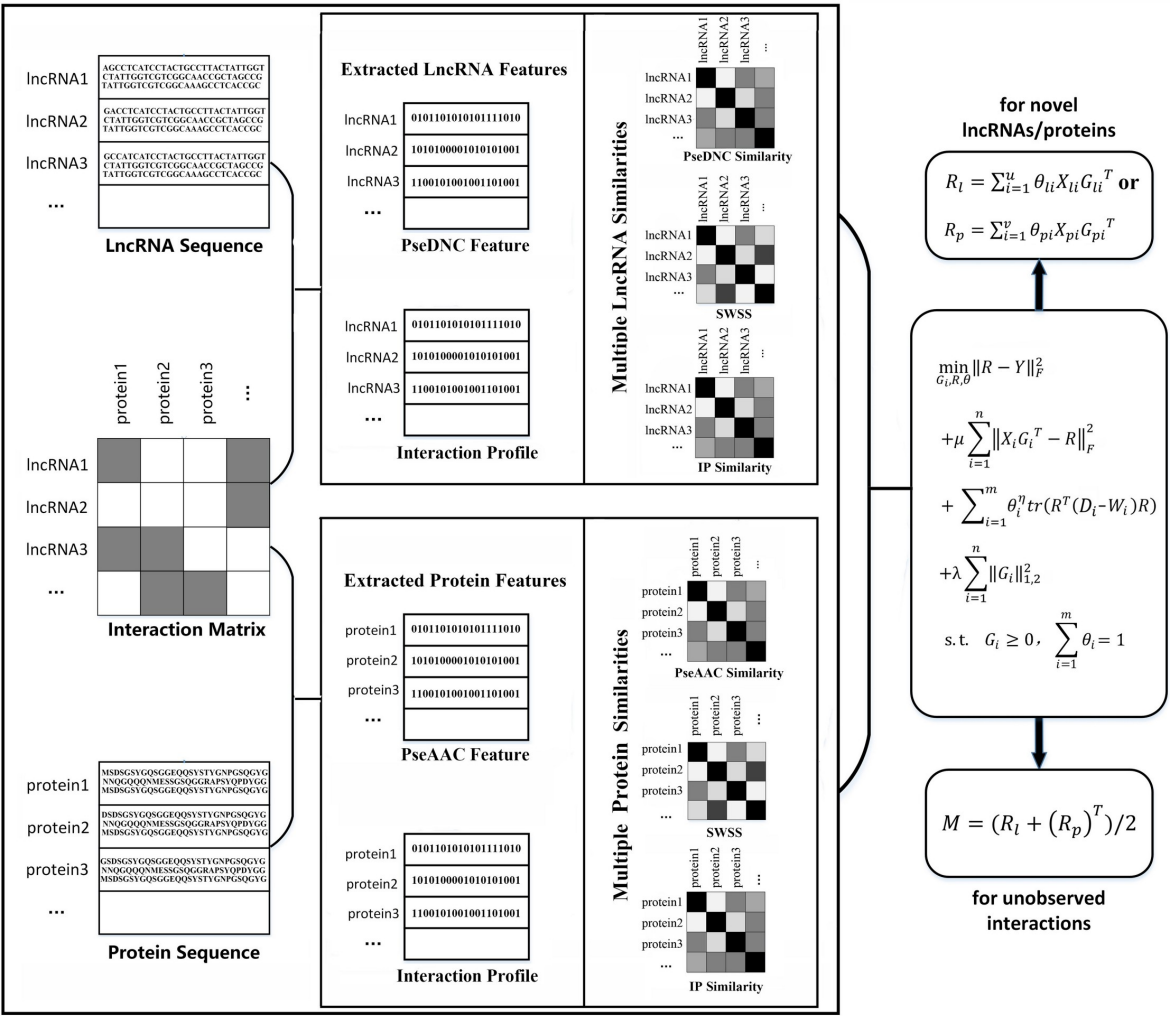
The flowchart of SFPEL-LPI for predicting lncRNA-protein interactions.

Combining various features or fusing various features can usually lead to high-accuracy models [52–58]. We have *n* features for lncRNAs (or proteins), denoted as *n* feature matrices ${\left\{X_{i}\right\}}_{i=1}^{n}$, and have *m* types of similarities for lncRNAs (or proteins), denoted as *m* similarity matrices ${\left\{W_{i}\right\}}_{i=1}^{m}$. The predicted lncRNA-protein interaction matrix is denoted as *R*. The known lncRNA-protein interaction matrix is denoted as *Y*. The flowchart of the feature projection ensemble learning method SFPEL-LPI is shown in Fig 1.

#### Objective function

First, lncRNA (or protein) feature matrices ${\left\{X_{i}\right\}}_{i=1}^{n}$ are respectively projected to the predicted lncRNA-protein interaction matrix *R* by using the projection matrices ${\left\{G_{i}\right\}}_{i=1}^{n}$. We estimate the projection matrices ${\left\{G_{i}\right\}}_{i=1}^{n}$ for features by minimizing the squared error between their products and the predicted lncRNA-protein interaction matrix *R*. So we have:

$$\begin{array}{c} \underset{G_{i}}{min}\sum_{i=1}^{n}{\left\|X_{i}{G_{i}}^{T}-R\right\|}_{F}^{2}\\ s.t.G_{i}\geq 0 \end{array}$$ (3)

where ${\left\|∙\right\|}_{F}^{2}$ is the Frobenius norm, and the projection matrices ${\left\{G_{i}\right\}}_{i=1}^{n}$ are required to be nonnegative.

Then, we introduce the $l_{1,2}$-norm regularization term of ${\left\{G_{i}\right\}}_{i=1}^{n}$ to ensure the smoothness of the projection matrices. The predicted matrix *R* should be approximated to the known interaction matrix *Y*. We can have

$$\begin{array}{c} \underset{G_{i},R}{min}\;{\left\|R-Y\right\|}_{F}^{2}+\mu \sum_{i=1}^{n}{\left\|X_{i}{G_{i}}^{T}-R\right\|}_{F}^{2}+\lambda \sum_{i=1}^{n}{\left\|G_{i}\right\|}_{1,2}^{2}\\ s.t.G_{i}\geq 0 \end{array}$$ (4)

where λ is the regularization coefficient, and *μ* is a trade-off parameter. ${\left\|G_{i}\right\|}_{1,2}=\sqrt{\sum_{k}{\left(\sum_{l}\left|g_{k,l}^{i}\right|\right)}^{2}}$.

Local structure of data can be maintained effectively through constructing a weighted graph or a similarity graph on a scatter of data points. For example, Xu et al. [59] introduced the manifold regularization term to preserve the visual feature manifold structure. Nie et al. [60], Bai et al. [61], Cai et al. [62,63] adopted graph Laplacian matrix to keep the graph’s local structure. Moreover, the Studies [34,64–67] revealed that the combination of multiple similarities helps to improve performances. Inspired by pioneer work, we define a novel ensemble graph Laplacian regularization:

$$\sum_{i=1}^{m}\theta_{i}^{\eta }tr(R^{T}(D_{i}-W_{i})R)$$ (5)

where *D*_*i*_ is a diagonal matrix whose diagonal elements are corresponding row sums of *W*_*i*_, and *θ* = [*θ*_1_,*θ*_2_,⋯,*θ*_*i*_,⋯,*θ*_*m*_] is a weight vector which is introduced to control the contribution of different graph Laplacian regularizations, and *tr*(∙) is the trace of a matrix. *η*>1 is the exponent of *θ*, which ensures that all graph Laplacian regularizations contribute effectively for the maintaining of graph local structures.

By combining (4) and (5), we obtain the objective function of SFPEL-LPI:

$$\begin{array}{c} \underset{G_{i},R,\theta }{min}{\left\|R-Y\right\|}_{F}^{2}+\mu \sum_{i=1}^{n}{\left\|X_{i}{G_{i}}^{T}-R\right\|}_{F}^{2}+\sum_{i=1}^{m}\theta_{i}^{\eta }tr(R^{T}(D_{i}-W_{i})R)+\lambda \sum_{i=1}^{n}{\left\|G_{i}\right\|}_{1,2}^{2}\\ s.t.G_{i}\geq 0,\sum_{i}\theta_{i}=1 \end{array}$$ (6)

We introduce the Lagrangian function (Lf) to solve the optimization problem in (6),

$$Lf={\left\|R-Y\right\|}_{F}^{2}+\mu \sum_{i=1}^{n}{\left\|X_{i}{G_{i}}^{T}-R\right\|}_{F}^{2}+\sum_{i=1}^{m}\theta_{i}^{\eta }tr(R^{T}(D_{i}-W_{i})R)+\lambda \sum_{i=1}^{n}{\left\|G_{i}\right\|}_{1,2}^{2}-\delta (\sum_{i=1}^{m}\theta_{i}-1)-\sum_{i=1}^{n}tr(\Gamma_{i}G_{i})$$

We calculate the partial derivatives of above function with respect to *R*, *G*_*i*_ and *θ*_*i*_, and obtain the update rules about *R*, *θ*_*i*_ and *G*_*i*_ (proof and deduction are provided in S1 File):

$$R={\left(\sum_{i=1}^{m}\theta_{i}^{\eta }(D_{i}-W_{})+(1+n\mu )I\right)}^{-1}(Y+\mu \sum_{i=1}^{n}X_{i}{G_{i}}^{T})$$ (7)

$$\theta_{i}=\frac{{\left(\frac{1}{tr(R^{T}(D_{i}-W_{i})R)}\right)}^{\frac{1}{\eta -1}}}{\sum_{i}^{m}{\left(\frac{1}{tr(R^{T}(D_{i}-W_{i})R)}\right)}^{\frac{1}{\eta -1}}}$$ (8)

$$G_{i}=G_{i}⨀\sqrt{\frac{{G_{i}(\mu {X_{i}}^{T}X_{i}+\lambda ee^{T})}^{+}+\mu {\left(R^{T}X_{i}\right)}^{-}}{{G_{i}(\mu {X_{i}}^{T}X_{i}+\lambda ee^{T})}^{-}+\mu {\left(R^{T}X_{i}\right)}^{+}}}$$ (9)

where *e* is a column vector with all elements equal to 1, and has the same column dimensions as *X*_*i*_. ⨀ denotes element-wise multiplication (also well known as Hadamard product), and the division in (9) is element-wise division. We separate the positive and negative parts of matrix *A* as

$$A^{+}=\frac{\left(|A|+A\right)}{2},A^{-}=\frac{\left(|A|-A\right)}{2}$$ (10)

Thus, we update *R*, *G*_*i*_ and *θ*_*i*_ based on (7), (8) and (9) alternatively until convergence.

#### Algorithms

Following the method proposed in the Section “Objective function”, SFPEL-LPI can predict unobserved interactions between known lncRNAs and proteins. First, based on the lncRNA’s features, similarities and lncRNA-protein interactions, the prediction matrix *R*_*l*_ could be obtained. Similarly, using protein’s features, similarities and protein-lncRNA interactions, the prediction matrix *R*_*p*_ could be calculated. Then, SFPEL-LPI integrates the predictions based on lncRNAs and proteins as *M* = (*R*_*l*_+(*R*_*p*_)^*T*^)/2. Therefore, the unobserved interactions are scored in the corresponding entries of *M*. Algorithm 1 describes how SFPEL-LPI predicts unobserved associations between known lncRNAs and known proteins.

In addition, SFPEL-LPI could also be applied to predict proteins (or lncRNAs) interacting with new lncRNAs (or proteins). After using Algorithm 1 to train the model, the projection matrix and the weighting parameters of lncRNA’s features as well as protein’s features: *G*_*lu*_, *G*_*lv*_, *θ*_*lu*_ and *θ*_*lv*_ could be obtained. Then, we can use the features of new lncRNAs (or proteins) and the trained parameters to predict their predictions. Algorithm 2 describes how SFPEL-LPI finishes this task.

**Algorithm 1**: **Predicting unobserved associations between known lncRNAs and known proteins by SFPEL-LPI**.

**Input:** observed lncRNA-protein interaction matrix, *Y*_*l*_; observed protein-lncRNA interaction matrix, *Y*_*p*_ = *Y*_*l*_^*T*^; lncRNA feature matrices, {*X*_*l*1_,*X*_*l*2_,…,*X*_*ln*_}; protein feature matrices, {*X*_*p*1_,*X*_*p*2_,…,*X*_*pn*_}; lncRNA normalized similarity matrices, {*W*_*l*1_,*W*_*l*2_,…,*W*_*lm*_}; protein normalized similarity matrices, {*W*_*p*1_,*W*_*p*2_,…,*W*_*pm*_}; regularization parameter, *μ*>0,*λ*>0; exponent parameter, *η*>1;

**Output:** lncRNA-protein interaction prediction matrix, *M*; predicted lncRNA-protein interaction matrix, *R*_*l*_; predicted protein-lncRNA interaction matrix, *R*_*p*_; projection matrices of lncRNA features {*G*_*l*1_,*G*_*l*2_,…,*G*_*ln*_}; projection matrices of protein features {*G*_*p*1_,*G*_*p*2_,…,*G*_*pn*_}; weighting parameters of lncRNA similarity matrices, {*θ*_*l*1_,*θ*_*l*2_,…,*θ*_*lm*_}; weighting parameters of protein similarity matrices, {*θ*_*p*1_,*θ*_*p*2_,…,*θ*_*pm*_};

**Initialize:**

**for** each *i*(1≤*i*≤*n*)

    initialize *G*_*li*_, *G*_*pi*_ with random values on interval [0,1];

**end for**

**for** each *i*(1≤*i*≤*m*)

    initialize *θ*_*li*_, *θ*_*pi*_ as 1/*m*;

**end for**

**repeat**

    update *R*_*l*_ via (7) with fixing ${\left\{G_{li}\right\}}_{i=1}^{n}$, ${\left\{\theta_{li}\right\}}_{i=1}^{m}$;

    **for** each *i*(1≤*i*≤*n*)

        update *G*_*li*_ via (8) with fixing *R*_*l*_;

    **end for**

    **for** each *i*(1≤*i*≤*m*)

        update *θ*_*li*_ via (9) with fixing *R*_*l*_:

    **end for**

**until Converges;**

**repeat**

    update *R*_*p*_ via (7) with fixing ${\left\{G_{pi}\right\}}_{i=1}^{n}$, ${\left\{\theta_{pi}\right\}}_{i=1}^{m}$;

    **for** each *i*(1≤*i*≤*n*)

        update *G*_*pi*_ via (8) with fixing *R*_*p*_;

    **end for**

    **for** each *i*(1≤*i*≤*m*)

        update *θ*_*pi*_ via (9) with fixing *R*_*p*_:

    **end for**

**until Converges;**

*M* = (*R_l_*+(*R_p_*)^*T*^)/2

**Return** *M*

**Algorithm 2**: **Predicting interacting proteins (or lncRNAs) for new lncRNAs (or proteins) by SFPEL-LPI**

**Input:** feature matrices for new lncRNAs, {*X*_*l*1_,*X*_*l*2_,…,*X*_*lu*_} (or feature matrices for new proteins, {*X*_*p*1_,*X*_*p*2_,…,*X*_*pv*_}); projection matrices of lncRNA features {*G*_*l*1_,*G*_*l*2_,…,*G*_*lu*_} (or projection matrices of protein features {*G*_*p*1_,*G*_*p*2_,…,*G*_*pv*_}); weighting parameters of lncRNA similarity matrices, {*θ*_*1*1_,*θ*_*l*2_,…,*θ*_*lu*_} (or weighting parameters of protein features, {*θ*_*p*1_,*θ*_*p*2_,…,*θ*_*pv*_}); (${\left\{G_{li}\right\}}_{i=1}^{u}$, ${\left\{\theta_{li}\right\}}_{i=1}^{u}$ or ${\left\{G_{pi}\right\}}_{i=1}^{v},\;{\left\{\theta_{pi}\right\}}_{i=1}^{v}$ are obtained by Algorithm 1);

**Output:** predicted lncRNA-protein interaction matrix, $R_{l}=\sum_{i=1}^{u}\theta_{li}X_{li}{G_{li}}^{T}$ (or predicted protein-lncRNA interaction matrix, $R_{p}=\sum_{i=1}^{v}\theta_{pi}X_{pi}{G_{pi}}^{T}$);

## Results

### Evaluation metrics

We adopt five-fold cross validation to evaluate the performances of prediction models. The proposed method SFPEL-LPI can predict unobserved interactions between known lncRNAs and known proteins, and also can make predictions for new lncRNAs (or proteins). In predicting unobserved lncRNA-protein interactions, all known lncRNA-protein interactions are randomly split into five subsets with equal size. Each time, four subsets are combined as training set and the remaining one subset is used as the testing set. In predicting proteins interacting with new lncRNAs, all known lncRNAs are split into five subsets with equal size. The model is constructed based on the lncRNAs in training set and their interactions with all proteins, and then is used to predict proteins interacting with testing lncRNAs. Similarly, we evaluate the performances of models in predicting lncRNAs interacting with new proteins. Hence, we introduce notations for above mentioned cross validation settings. *CV*_*lp*_: known lncRNA-protein interactions are split into five folds in predicting unobserved interactions. *CV*_*l*_: known lncRNAs are split into five folds in predicting interactions for new lncRNAs. *CV*_*p*_: known proteins are split into five folds in predicting interactions for new proteins.

The area under ROC curve (AUC) and the area under precision-recall curve (AUPR) are popular metrics for evaluating prediction models. Since known lncRNA-protein interactions are much less than non-interacting lncRNA-protein pairs, we adopt AUPR as the primary metric, which punishes false positive more in the evaluation process[68,69]. Moreover, we adopt several binary classification metrics, i.e. recall (REC), accuracy (ACC), precision (PR) and F1-measure (F1).

### Parameter setting

SFPEL-LPI has three parameters: *μ*, λ and *η*. *μ* is a parameter for the error between projected interactions and predicted lncRNA-protein interactions; *λ* controls the contribution of projection matrix; *η* describes strength of different similarity measures.

**Fig 2:**
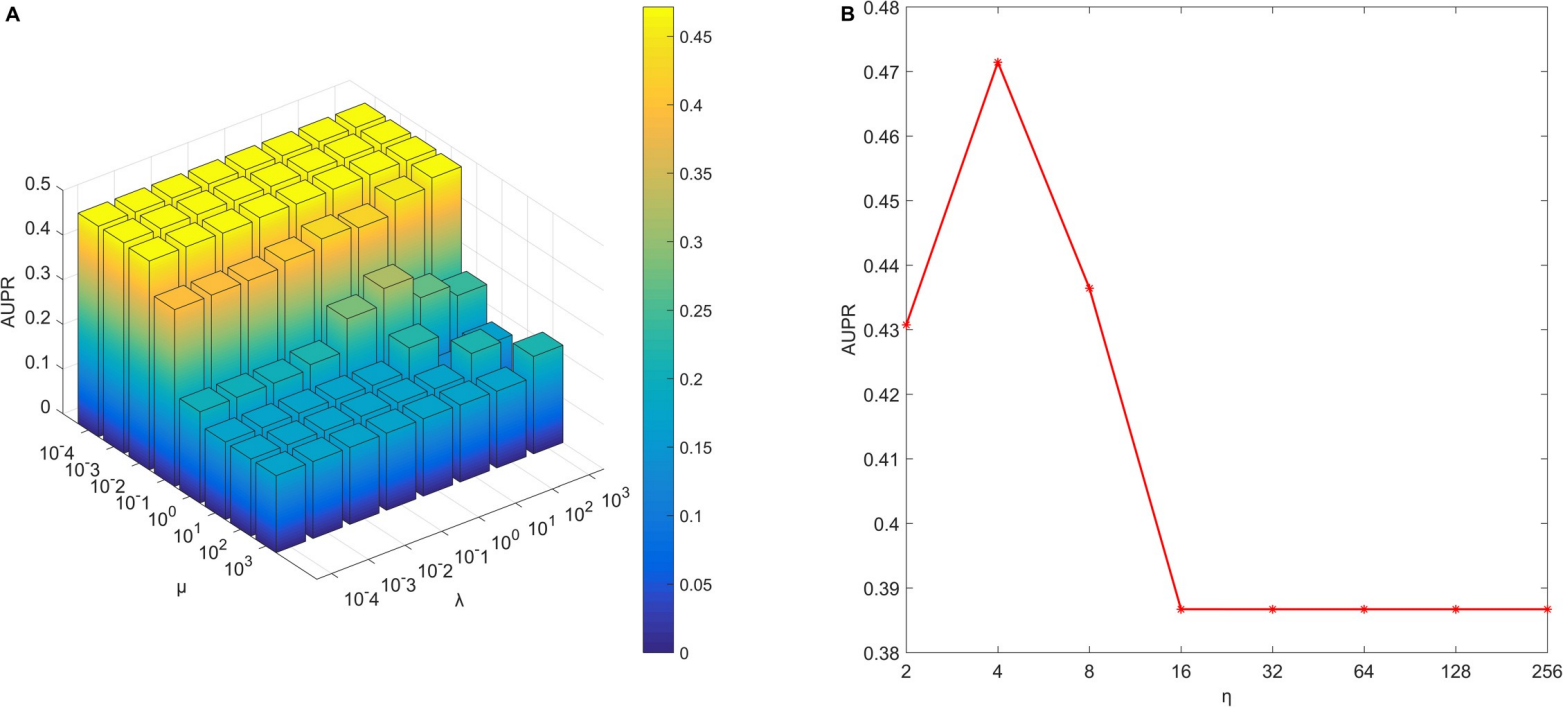
The influence of parameters on AUPR of models. (A) Fix the parameter *η* = 2^2^, and evaluate the influence of parameters *μ* and λ. (B) Fix the parameter *μ* = 10^−1^, *λ* = 10^3^, and evaluate the influence of parameter *η*.

To test influence of parameters, we consider all combinations of parameters *μ*∈{10^−4^,10^−3^,10^−2^,10^−1^,10^0^,10^1^,10^2^,10^3^}, *μ*∈{10^−4^,10^−3^,10^−2^,10^−1^,10^0^,10^1^,10^2^,10^3^} and *η*∈{2^1^,2^2^,2^3^,2^4^,2^5^,2^6^,2^7^,2^8^}. We build SFPEL-LPI models by using different parameters, and implement five-fold cross validation *CV*_*lp*_ to evaluate SFPEL-LPI models. SFPEL-LPI produces the best AUPR score of 0.473 when *μ* = 10^−3^, λ = 10^−4^ and *η* = 2^2^. Then, we fix the parameter *η* = 2^2^, and evaluate the influence of *μ* and λ. As shown in Fig 2A, *μ* greatly influences the performance of SFPEL-LPI, and a smaller value for *μ* is likely to produce better result. Further, we fix the parameters *μ* = 10^−3^ and λ = 10^−4^ and test the influence of *η*. As illustrated in Fig 2B, the performances of SFPEL-LPI decrease as *η* increases, and then remain unchanged after a threshold.

**Fig 3:**
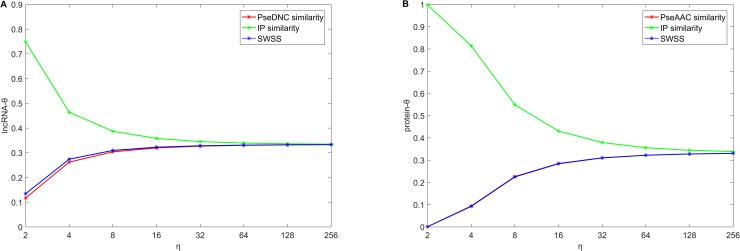
(A) The relationship between *η* and *θ*_*lncRNA*_. (B) The relationship between *η* and *θ*_*protein*_.

The parameter *η* is the index of similarity weights, and could control the relative contributions of different similarities. When fixing *μ* = 10^−3^ and λ = 10^−4^, we analyze the relation between *η* and lncRNA similarity measures *θ*_*lncRNA*_ (or protein similarity measures *θ*_*protein*_). As shown in Fig 3, similarities usually make different contributions to SFPEL-LPI models, and interaction profile similarities usually make more contributions than other similarities. With increase of *η*, different similarities are likely to make equal contributions.

Based on above discussion, we adopt *μ* = 10^−3^, λ = 10^−4^ and *η*= 2^2^ for SFPEL-LPI in the following studies.

### Performances of SFPEL-LPI

SFPEL-LPI can predict unobserved lncRNA-protein interactions between known lncRNAs and known proteins, and also can make predictions for new lncRNAs (or proteins). For different tasks, we adopt different evaluation schemes to split instances and implement five-fold cross validation under settings: *CV*_*lp*_, *CV*_*l*_ and *CV*_*p*_.

**Fig 4:**
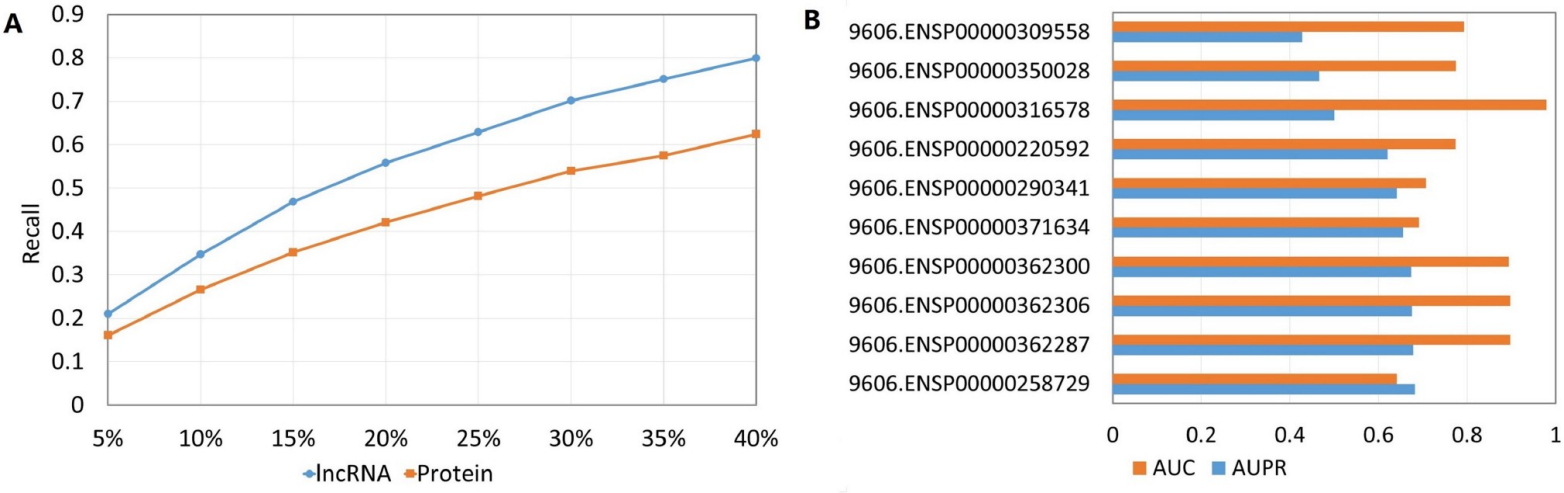
(A) The average recalls in predicting new lncRNAs (or proteins) at different top-ranked percentiles under CV_*l*_ or CV_*p*_. (B) The AUC value and AUPR value of predicting interacting lncRNAs for selected new proteins.

Table 1 displays AUPR scores and AUC scores of SFPEL-LPI evaluated by CV_*lp*_, CV_*l*_ and CV_*p*_. According to previous studies [70–72], a prediction model that can accurately recover the true interacting proteins (or lncRNAs) is usually desired and useful for the wet experimental validation. Thus, we calculate the proportion of correctly predicted true interactions at different top-ranked percentiles under CV_*l*_ or CV_*p*_. A new matric “recall @ top-ranked k %” is defined as the fraction of true interacting proteins (or lncRNAs) that are retrieved in the list of top-ranked k% predictions for a lncRNA (or protein). In Fig 4A, SFPEL-LPI performs effectively in predicting proteins (or lncRNAs) interacting with new lncRNAs (or proteins). The reason why the performances of predicting lncRNAs interacting with new proteins is not as well as the performances of predicting proteins interacting with new lncRNAs is that the number of lncRNAs (990) in our dataset is much more than the number of proteins (27). Consequently, less information is used to train SFPEL-LPI models.

**Table 1:** Performances of SFPEL-LPI for predicting lncRNA-protein interactions.

Cross Validation	AUPR	AUC	PRE	REC	ACC	F1
CV_*lp*_	0.473	0.920	0.449	0.495	0.960	0.470
CV_*l*_	0.490	0.823	0.449	0.552	0.823	0.493
CV_*p*_	0.339	0.656	0.325	0.476	0.749	0.375

To further test capability of SFPEL-LPI for new proteins, we randomly select ten proteins to conduct experiments. In each experiment, a protein is used as the testing protein, and the model is constructed based on other proteins, all lncRNAs and their associations, and then predict lncRNAs interacting with the testing protein. AUC scores and AUPR scores are calculated based on the results for each protein. As shown in Fig 4B, SFPEL-LPI produces the AUPR values greater than 0.6 and the AUC values greater than 0.7 for most proteins, indicating great potential of predicting lncRNAs interacting with new proteins.

### Comparison with state-of-the-art prediction methods

Several state-of-the-art computational methods have been proposed to predict lncRNA-protein interactions. Here, we adopt RWR[17], LPBNI[17], KATZLGO[19], LPI-ETSLP [20] and LPLNP [21] for comparison. RWR implemented random walk with restart to predict lncRNA-protein interactions. LPBNI constructed a lncRNA-protein bipartite network based on known lncRNA-protein interactions, and then predicted lncRNA-protein interactions by using the resource allocation algorithm. KATZLGO constructed a heterogeneous network based on lncRNA-lncRNA similarity, lncRNA-protein interactions and protein-protein similarity, and then adopted KATZ measure to calculate distances between lncRNAs and proteins in the network. LPI-ETSLP calculated lncRNA-lncRNA similarity and protein-protein similarity based on pairwise sequence Smith-Waterman scores, and then built semi-supervised link prediction classifier based on these similarities. LPNLP calculated three lncRNA-lncRNA similarities and two protein-protein similarities by using linear neighborhood similarity measure, and implemented label propagation to develop the integrated models.

First, we respectively build different prediction models based on the benchmark dataset. The benchmark methods were designed to predict unobserved interaction between know lncRNAs and know proteins. Therefore, we implement these methods and mainly evaluate their performances in predicting unobserved interactions under *CV*_*lp*_. As shown in Table 2, the AUPR values of RWR, LPBNI, KATZLGO, LPI-ETSLP, LPLNP and SFPEL-LPI are 0.236, 0.330, 0.286, 0.322, 0.459, 0.473, and AUC values are 0.850, 0.856, 0.760, 0.889, 0.910 and 0.920, respectively. SFPEL-LPI outperforms these five methods, and makes 100.4%, 43.3%, 65.4%, 46.9%, 3.1% improvements in terms of AUPR scores and 8.2%, 7.5%, 21.1%, 3.5%, 1.1% improvements in terms of AUC scores when compared with five benchmark methods. Though SFPEL-LPI produces slightly better performances than LPLNP in terms of AUPR and AUC, LPLNP utilizes more information than SFPEL-LPI for modeling. To be more specific, LPLNP uses three lncRNA features (“interaction profile”, “expression profile”, “sequence composition”) and two protein features (“interaction profile”, “CTD”), while SFPEL-LPI only used lncRNA sequences, protein lncRNAs and known lncRNA-protein interactions.

**Table 2:** Performances of prediction methods on the benchmark dataset.

Method	AUPR	AUC	PRE	REC	ACC	F1
RWR	0.236	0.850	0.245	0.391	0.935	0.299
LPBNI	0.330	0.856	0.413	0.370	0.958	0.386
KATZLGO	0.286	0.760	0.354	0.348	0.954	0.350
LPI-ETSLP	0.322	0.889	0.374	0.423	0.953	0.394
LPLNP	0.459	0.910	0.523	0.404	0.965	0.453
SFPEL-LPI	0.473	0.920	0.449	0.495	0.960	0.470

We conduct 20 runs of five-fold cross validation to evaluate methods, and take the paired t-test to analyze difference between SFPEL-LPI and benchmark methods. Table 3 demonstrates that SFPEL-LPI produces significantly better results than state-of-the-art methods in terms of AUC and AUPR.

**Table 3:** Difference between SFPEL-LPI and benchmark methods tested by Paired t-test in terms of AUPR and AUC.

AUPR				
RWR	LPBNI	KATZLGO	LPI-ETSLP	LPLNP
6.35E-37	3.55E-32	1.91E-34	3.37E-31	4.38E-12
		AUC		
RWR	LPBNI	KATZLGO	LPI-ETSLP	LPLNP
1.43E-26	5.94E-28	8.15E-34	1.59E-31	1.37E-19

The computational complexity is important for a computational method. To test the efficiency of SFPEL-LPI, we repeat 5-fold cross validation 20 times and compare running time of different methods on a PC with an Intel i7 7700k CPU and 16GB RAM. SFPEL-LPI costs the reasonable running time (29.42s) when compared with RWR (25.83s), LPBNI (4.01s), KATZLGO (4.36s), LPI-ETSLP (4.56s) and LPLNP (1337.64s).

**Fig 5:**
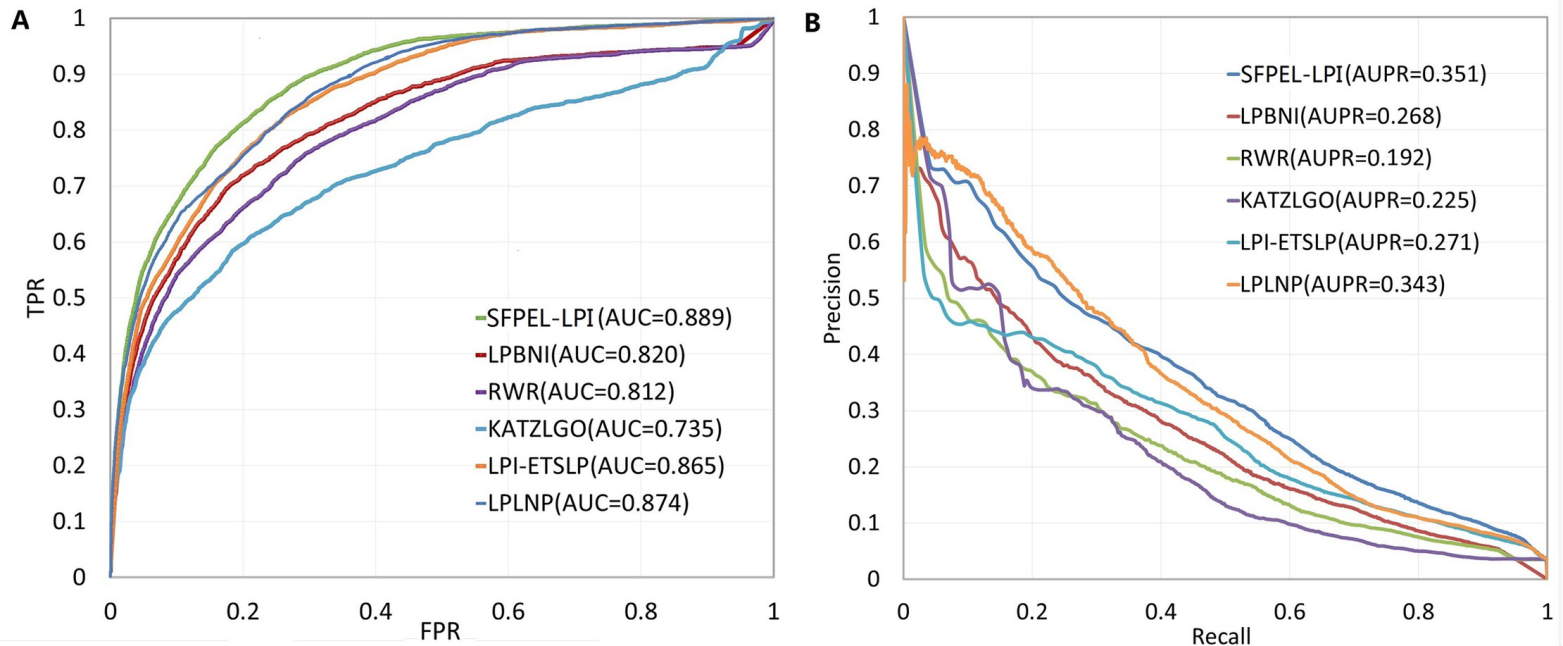
Performance of different methods on the perturbed dataset. (A) ROC curves. (B) PR curves.

Further, we randomly perturb all known lncRNA-protein interactions to test the robustness of prediction methods. To be more specific, we randomly remove 5% of known lncRNA-protein interactions and add the same number of inexistent interactions, and then compile the perturbed dataset. We build different prediction models based on the perturbed dataset and evaluate their performances. Clearly, data perturbation brings noise, and decreases the performances of prediction models. As displayed in Fig 5, AUC scores of RWR, LPBNI, KATZLGO, LPI-ETSLP, LPLNP, SFPEL-LPI are 0.812, 0.820, 0.735, 0.865, 0.874 and 0.889; AUPR scores are 0.192, 0.268, 0.225, 0.271, 0.343 and 0.351. Although prediction models produce lower performances than that in Table 2, SFPEL-LPI still produces satisfying results, and outperforms RWR, LPBNI, KATZLGO, LPI-ETSLP and LPLNP.

### Independent experiments

**Fig 6:**
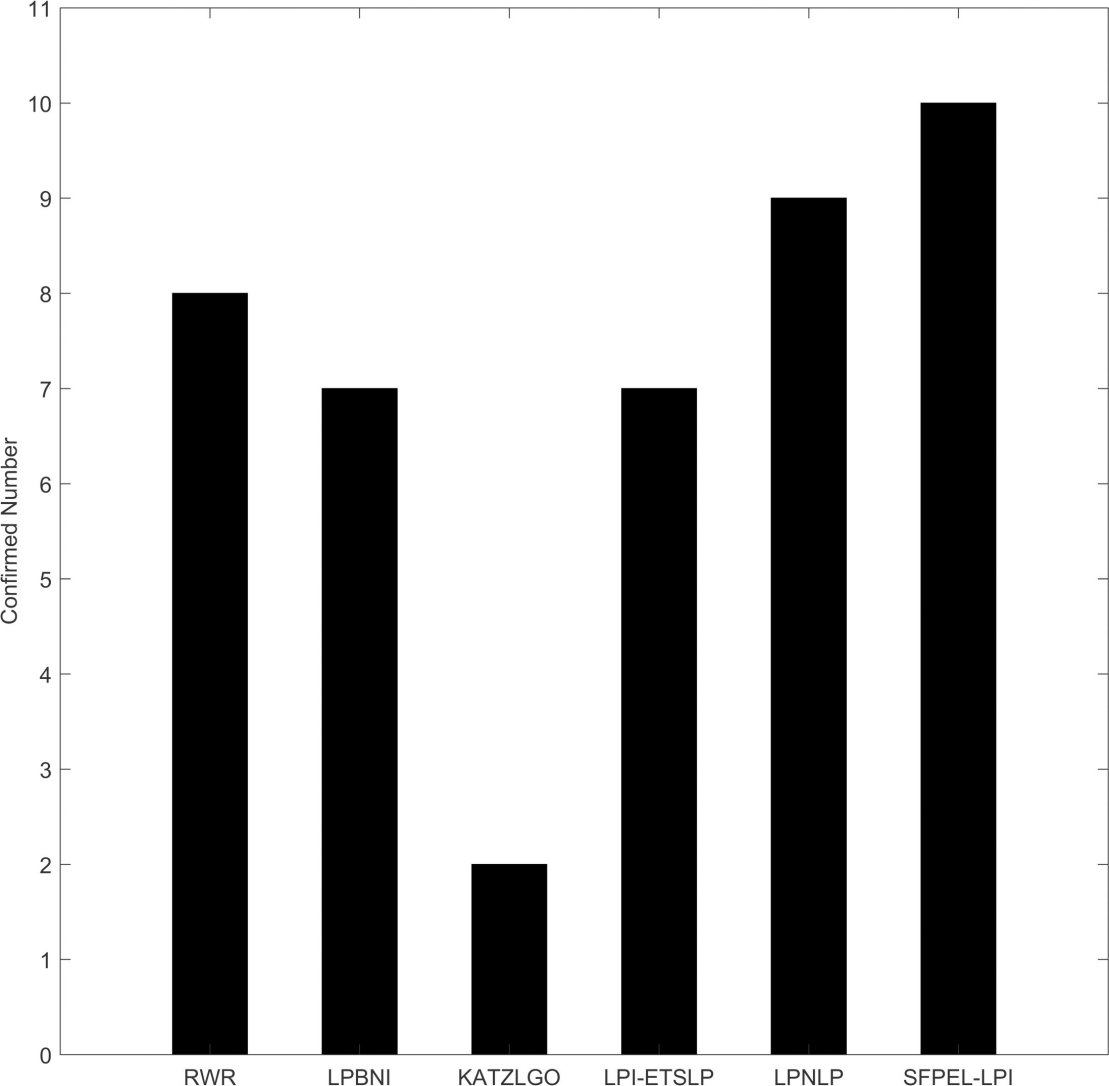
The number of confirmed lncRNA-protein interactions in top 20 predictions of different methods.

Here, we conduct independent experiments to evaluate the practical ability of SFPEL-LPI. As described in Section “Dataset”, NPInter v2.0 dataset was compiled from the V2.0 edition of NPInter database. NPInter database has been updated to V3.0 edition, and contains newly discovered lncRNA-protein interactions. Therefore, we train the prediction model based on the NPInter v2.0 dataset and predict new lncRNA-protein interactions, and then check up on predictions in the NPInter database. Fig 6 shows the number of confirmed interactions in top 20 predictions of all methods. Clearly, SFPEL-LPI finds out more interactions than benchmark methods. In addition, we observe that most of novel interactions identified by SFPEL-LPI have low ranks in the predictions of other benchmark methods, indicating that SFPEL-LPI can find out interactions ignored by these methods. Top predictions and their ranks are provided in S1 Table.

### Web server

We develop a web server based on SFPEL-LPI to facilitate the lncRNA-protein interaction prediction, available at http://www.bioinfotech.cn/SFPEL-LPI/. Users can input lncRNA sequences (or protein sequences) or upload a text file with FASTA-formatted lncRNA sequences (or protein sequences) for prediction, and freely download the results and visualize the predicted lncRNA-protein interactions. Moreover, gene ontology (GO) terms of proteins are annotated for indicating lncRNAs’ functions.

**Fig 7:**
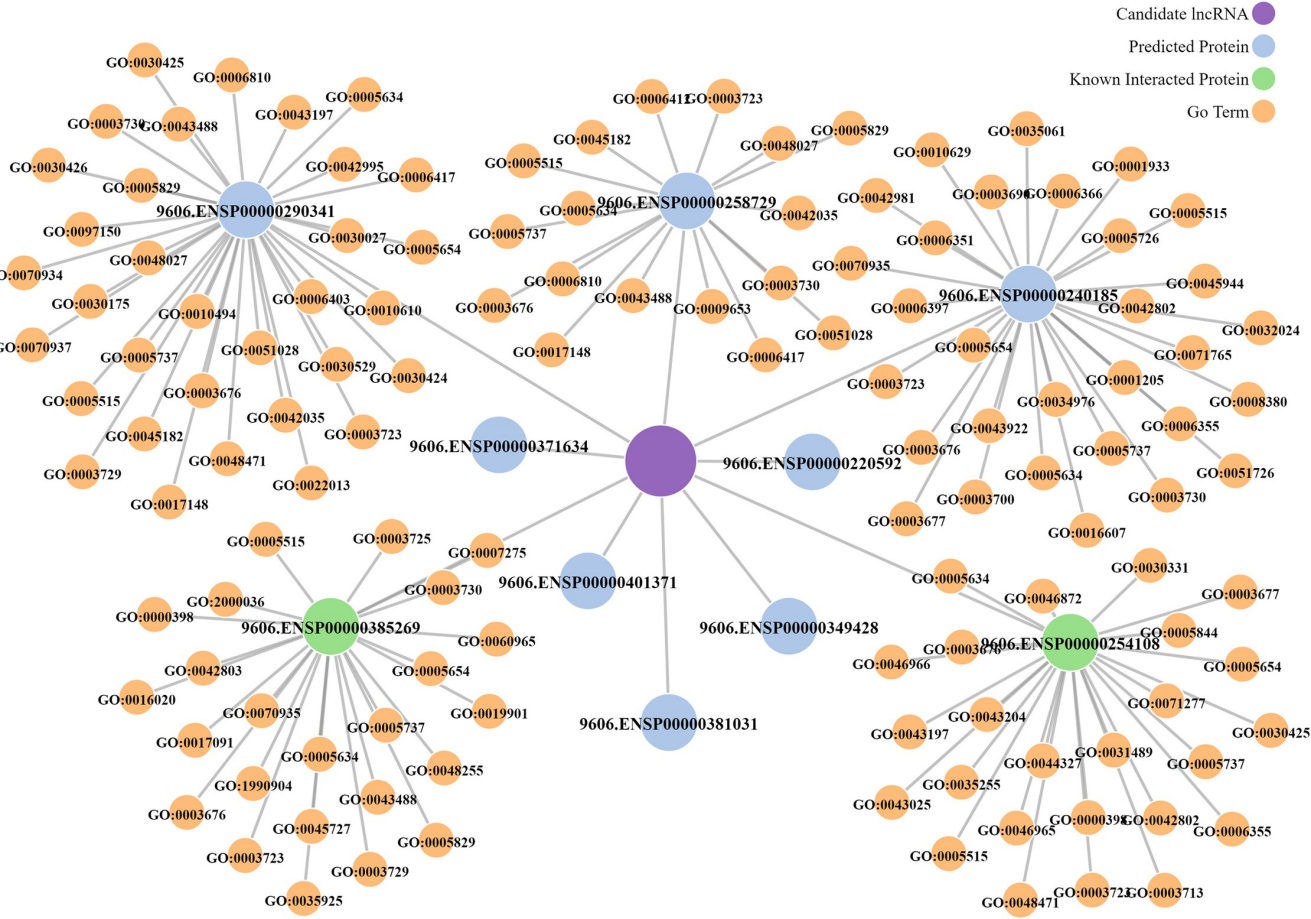
Visualization of top 10 predicted interacting proteins for the lncRNA: “NONHSAT041930”. Purple node stands for the lncRNA. Navy blue nodes indicate the predicted interacting proteins, and green nodes represent proteins that have observed interactions with the lncRNA. Moreover, we map the corresponding GO Terms (Orange nodes) of each interacting protein from QuickGO database (https://www.ebi.ac.uk/QuickGO/).

Fig 7 displays the top 10 predictions for the lncRNA “NONHSAT041930”. “NONHSAT041930” named OIP5-AS1 (OIP5 antisense RNA 1), is a mammalian lncRNA that is abundant in the cytoplasm [73]. OIP5-AS1 has gained wide attention. In 2011, it was first identified to be involved in brain and eye development [74]. In 2016, Kim et al.[75] found that it can prevent HuR binding to target mRNAs and thus suppress the HuR-elicited proliferative phenotypes. Moreover, the lncRNA was found to interact with GAK mRNA, promoting GAK mRNA decay and hence reducing GAK protein levels and lowering cell proliferation [76]. Among top 10 predicted proteins interacting with OIP5-AS1, two proteins have already been known to have interactions with OIP5-AS1, which are included in the NPInter dataset. In addition, we find evidence from literature to support other six predicted proteins. For example, IGF2BP1, IGF2BP2, IGF2BP3, EWSR1 and TIA1 have already been examined to interact with OIP5-AS1 according to lncRNA-protein interacting data report [77]. Protein Argonaute 2 (AGO2) is required for proper nuclear migration, pole cell formation, and cellularization during the early stages of embryonic development. Several studies [75,78] showed that OIP5-AS1 is associated with AGO2. Moreover, annotated GO terms of predicted proteins indicate the function of the lncRNA OIP5-AS1: mRNA binding (GO: 0005845, GO: 0035925, GO: 0036002, GO: 0048027, GO: 0098808) and cell proliferation (GO:0022013). More details are provided in S2 Table. These encouraging instances demonstrate that the proposed method can successfully predict novel lncRNA-protein interactions.

**Fig 8:**
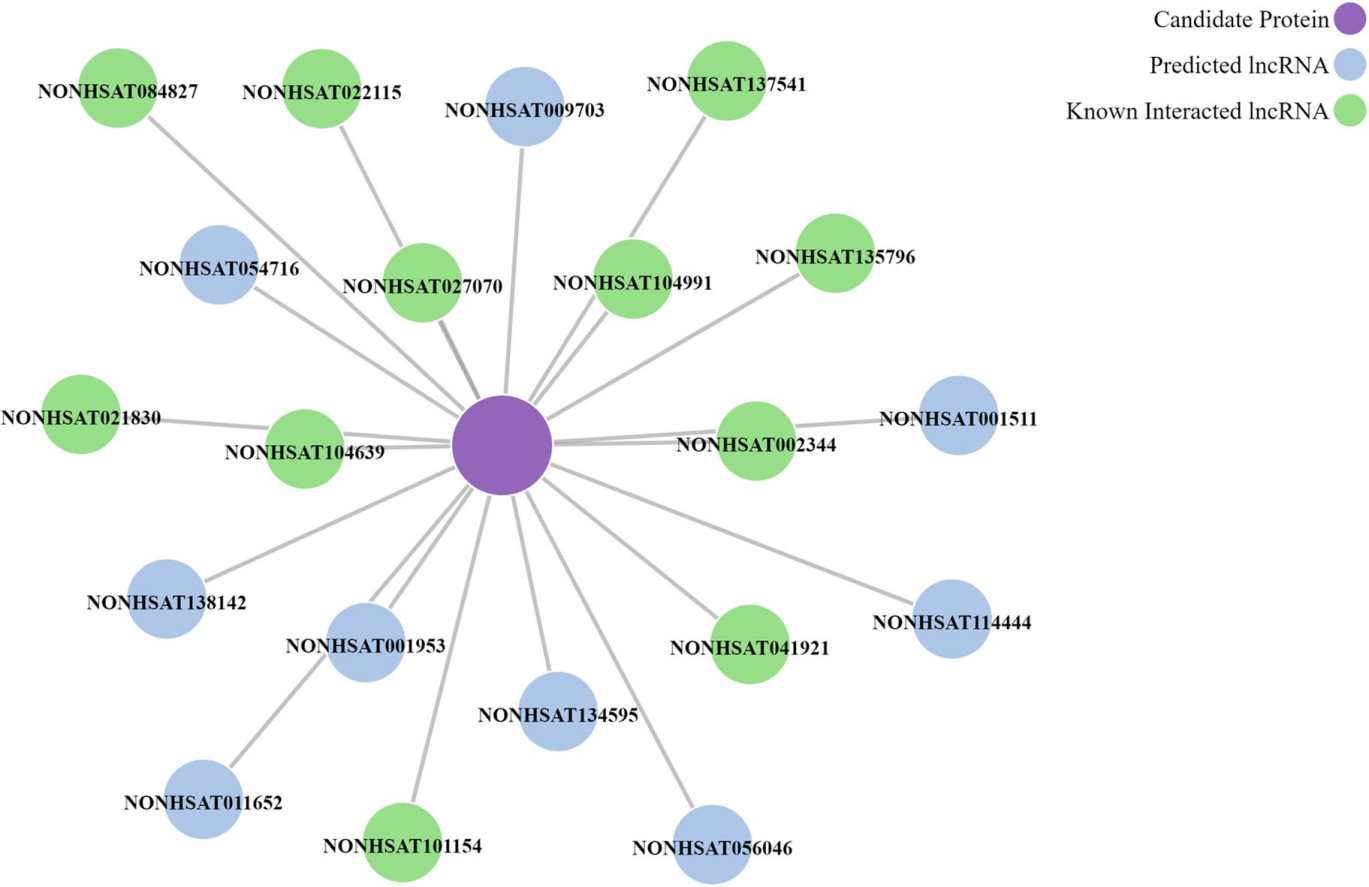
Visualization of top 20 predicted interacting lncRNAs of the protein: 9606.ENSP00000240185. Purple node stands for the protein. Navy blue nodes indicate the predicted interacting lncRNAs and green nodes represent lncRNAs that have observed interactions with the protein.

Moreover, the server can predict interacting lncRNAs for proteins. For example, top 20 interacting lncRNAs of the protein “9606.ENSP00000240185” are shown in the Fig 8, and details are provided in S3 Table.

## Discussion

This paper presents a novel lncRNA-protein interaction prediction method, namely sequence-based feature projection ensemble learning (SFPEL-LPI). The novelty of SFPEL-LPI comes from integrating sequence-derived features and similarities with a feature projection ensemble learning frame. Specifically, SFPEL-LPI only utilizes lncRNA sequences, protein sequences and known interactions to extract features, and calculates lncRNA-lncRNA similarities and protein-protein similarities. Since sequences are usually available for lncRNAs or proteins, SFPEL-LPI can make predictions for almost all lncRNA-protein pairs. Moreover, diverse information leads to the good performances of SFPEL-LPI.

To evaluate the performance of SFPEL-LPI, an extensive set of experiments were performed on the benchmark dataset under three CV setting: *CV*_*lp*_, *CV*_*l*_ and *CV*_*p*_, compared with state-of-the-art lncRNA-protein interaction prediction methods. The promising results validate efficacy of the proposed algorithm for predicting lncRNA-protein interactions, especially for the new lncRNAs or new proteins, which do not have known interactions. SFPEL-LPI outperforms five methods: RWR, LPBNI, KATZLGO, LPI-ETSLP, LPLNP, and makes 100.4%, 43.3%, 65.4%, 46.9%, 3.1% improvements in terms of AUPR scores. Further, we also analyze the running time of SFPEL-LPI and benchmark methods, and randomly perturb all known lncRNA-protein interactions to test the robustness of prediction methods. A web server is constructed to predict interacting proteins/lncRNAs for given lncRNAs/proteins. We adopt the lncRNA “NONHSAT041930” as an example to predict interacting proteins, and can find evidences to confirm novel lncRNA-protein interactions.

However, SFPEL-LPI still has several limitations. It has three parameters, and parameter tuning is time-consuming. In addition, known lncRNA-protein interactions are limited, and performances of SFPEL-LPI will be improved if more interactions are known.

## Supporting information

S1 File Proof and analysis of SFPEL-LPI. (PDF)

S2 File The data of SFPEL-LPI. (MAT)

S1 Table Top 20 predictions of SFPEL-LPI and their ranks in predictions of benchmark methods. (DOCX)

S2 Table Top 10 interacting proteins of LncRNA “NONHSAT041930” (OIP5-AS1) predicted by SFPEL-LPI. (DOCX)

S3 Table Top 20 interacting lncRNAs of protein “9606.ENSP00000240185” (TAR DNA-binding protein 43) predicted by SFPEL-LPI. (DOCX)
