# Supplementary material for: SFPEL-LPI: Sequence-based feature projection ensemble learning for predicting LncRNA-protein interactions
Source: PLoS Comput Biol. 2018 Dec 11;14(12):e1006616. doi: 10.1371/journal.pcbi.1006616 (PMC6331124; doi:10.1371/journal.pcbi.1006616)
Supplement: S1 File — (PDF) [file pcbi.1006616.s001.pdf]

1. Proof and Analysis of SFPEL-LPI

1.1 Solution to the objective function for SFPEL-LPI

The objective function for SFPEL-LPI:

$$\min_{G_i,R,\theta} \|R-Y\|_F^2 + \mu \sum_{i=1}^{n} \left\|X_i {G_i}^T - R\right\|_F^2 + \sum_{i=1}^{m} \theta_i^{\eta} tr(R^T(D_i - W_i)R) + \lambda \sum_{i=1}^{n} \|G_i\|_{1,2}^2 \quad (1)$$

$$\text{s.t.}\, G_i \geq 0, \sum_i \theta_i = 1$$

To solve the optimization problem in (1), we introduce the Lagrangian function:

$$Lf = \|R-Y\|_F^2 + \mu \sum_{i=1}^{n} \left\|X_i {G_i}^T - R\right\|_F^2 + \sum_{i=1}^{m} \theta_i^{\eta} tr(R^T(D_i - W_i)R) + \lambda \sum_{i=1}^{n} \|G_i\|_{1,2}^2 - \delta\left(\sum_{i=1}^{m} \theta_i - 1\right) - \sum_{i=1}^{n} tr(\Gamma_i G_i) \quad (2)$$

The partial derivatives of above function with respect to R, G_i and θ_i:

$$\frac{\partial L}{\partial R} = 2(R-Y) + 2\mu \sum_{i=1}^{n} \left(R - X_i {G_i}^T\right) + 2 \sum_{i=1}^{m} \theta_i^{\eta} (D_i - W_i) R \quad (3)$$

$$\frac{\partial L}{\partial G_i} = 2\mu\left({X_i}^T X_i {G_i}^T - {X_i}^T R\right) + 2\lambda e e^T {G_i}^T - {\Gamma_i}^T \quad (4)$$

$$\frac{\partial L}{\partial \theta_i} = \eta \theta_i^{\eta-1} tr(R^T(D_i - W_i)R) - \delta \quad (5)$$

Using the Karush–Kuhn–Tucker (KKT) conditions[1],

$$\begin{cases} \frac{\partial L}{\partial R} = 0, \frac{\partial L}{\partial G_i} = 0, \frac{\partial L}{\partial \theta_i} = 0 \\ \Gamma_i \geq 0 \\ \Gamma_i \odot G_i = 0 \\ \sum_{i=1}^{m} \theta_i = 1 \end{cases} \quad (6)$$

We can readily obtain the update rules about R, θ_i and G_i:

$$R = \left(\sum_{i=1}^{m} \theta_i^{\eta} (D_i - W_i) + (1 + n\mu) I\right)^{-1} \left(Y + \mu \sum_{i=1}^{n} X_i {G_i}^T\right) \quad (7)$$

$$\theta_i = \frac{\left(\frac{1}{tr(R^T(D_i - W_i)R)}\right)^{\frac{1}{\eta-1}}}{\sum_i^m \left(\frac{1}{tr(R^T(D_i - W_i)R)}\right)^{\frac{1}{\eta-1}}} \quad (8)$$

$$G_i = G_i \odot \frac{\mu R^T X_i}{\mu G_i {X_i}^T X_i + \lambda G_i e e^T} \quad (9)$$

where e is a column vector with all elements equal to 1, and has the same column dimension as X_i. $\odot$ denotes element-wise multiplication (also well known as Hadamard product), and the division in (9) is element-wise division. However, since R is not required to be non-negative, the update rule (9) fails to ensure that all elements of G_i are non-negative. To solve this problem, we follow the similar optimization rule in [2]:

$$G_i = G_i \odot \sqrt{\frac{G_i(\mu X_i{}^T X_i + \lambda ee^T)^+ + \mu(R^T X_i)^-}{G_i(\mu X_i{}^T X_i + \lambda ee^T)^- + \mu(R^T X_i)^+}} \tag{10}$$

where we separate the positive and negative parts of matrix A as

$$A^+ = \frac{(|A|+A)}{2}, A^- = \frac{(|A|-A)}{2} \tag{11}$$

Thus, we can update R, G_i and θ_i based on (7), (8) and (10) alternately until convergence.

1.2 Proof of convergence

Here, we discuss the convergence of update rules. As R is fixed, the objective function is equivalent to the following equation:

$$\min_{G_i} \mu \sum_{i=1}^n \left\|X_i G_i{}^T - R\right\|_F^2 + \lambda \sum_{i=1}^n \|G_i\|_{1,2}^2 \tag{12}$$

$$\text{s.t.} \, G_i \geq 0$$

For simplicity and generality, we reformulate the objective function ($G \in \{G_i\}_{i=1}^n,$):

$$O = tr(G(\mu X^T X + \lambda ee^T)G^T) - 2tr(\mu X^T R G) \tag{13}$$

where e is a column vector whose elements are set to 1.

As presented above, the update rule of G is:

$$G^{t+1} = G^t \odot \sqrt{\frac{G^t(\mu X^T X + \lambda ee^T)^- + \mu(R^T X)^+}{G^t(\mu X^T X + \lambda ee^T)^+ + \mu(R^T X)^-}} \tag{14}$$

Theorem 1: The value of objective function (13) decreases monotonically under the update rule (14).

To prove this theorem, an auxiliary function method is utilized as similar to [2]. Before giving the proof of the Theorem 1, some preparatory work is introduced, as well as the definition of auxiliary function is given and some useful lemmas are proved.

Preparatory Work: according to (11), the objective function (13) is rewritten as:

$$F(G) = tr(GPG^T) - 2tr(G^T Q) = tr(GP^+G^T) + 2tr(G^T Q^-) - tr(GP^-G^T) - 2\text{tr}(G^T Q^+) \tag{15}$$

where $P = \mu X^T X + \lambda ee^T, Q = \mu R^T X.$

Definition 1: $H(G, G^t)$ is called as auxiliary function for $F(G)$, if it satisfies the following conditions for any G:

$$\begin{cases} H(G, G^t) \geq F(G) \\ H(G, G) = F(G) \end{cases} \tag{16}$$

Lemma 1: For any matrices $A \in \mathbb{R}_+^{n\times n}, B \in \mathbb{R}_+^{k\times k}, S \in \mathbb{R}_+^{n\times k}, S' \in \mathbb{R}_+^{n\times k}$, with A and B symmetric, the following inequality holds [2]:

$$\sum_{i=1}^n \sum_{p=1}^k \frac{(AS'B)_{ip} S_{ip}^2}{S'_{ip}} \geq tr(S^T A S B) \tag{17}$$

Lemma 2: If $H(G, G^t)$ is an auxiliary function of $F(G)$, and a sequence $\{G^t\}$ satisfies:

$$G^{t+1} = \arg\min_G H(G, G^t) \tag{18}$$

$\{F(G^t)\}$ is a monotonic decreasing sequence.

Proof (Lemma2): According to the **Definition 1**, we have:

$$\begin{cases} F(G^{t+1}) \leq H(G^{t+1}, G^t) \\ H(G^t, G^t) = F(G^t) \end{cases} \tag{19}$$

Then, according to the prerequisite of Lemma 2, we have:

$$H(G^{t+1}, G^t) \leq H(G^t, G^t) \tag{20}$$

Thus,

$$F(G^{t+1}) \leq F(G^t) \tag{21}$$

Up to now, we have already proved that $\{F(G^t)\}$ is a monotonic decreasing sequence.

Lemma 3: The following function is an auxiliary function of $F(G)$ given by (15):

$$H(G, G^t) = \sum_{pq} \frac{(G^t P^+)_{pq} G_{pq}^2}{(G^t)_{pq}} + \sum_{pq} Q_{pq}^- \frac{(G^t)_{pq}^2 + G_{pq}^2}{(G^t)_{pq}} - \sum_{pqk} P_{qk}^- (G^t)_{pq} (G^t)_{pk} \left(1 + \log \frac{G_{pq} G_{pk}}{(G^t)_{pq} (G^t)_{pk}}\right) - 2 \sum_{pq} Q_{pq}^+ (G^t)_{pq} \left(1 + \log \frac{G_{pq}}{(G^t)_{pq}}\right) \tag{22}$$

Proof (Lemma3): According to the inequality (17) in **Lemma 1**, by setting $A = I, B = P^+, S = G$, $S' = G^t$, we obtain:

$$tr(GP^+G^T) \leq \sum_{pq} \frac{(G^t P^+)_{pq} G_{pq}^2}{(G^t)_{pq}} \tag{23}$$

Using the common inequality $2a \leq (a^2 + b^2)/b$ with $a, b > 0$, we have:

$$2tr(G^T Q^-) = 2 \sum_{pq} G_{pq} Q_{pq}^- \leq \sum_{pq} Q_{pq}^- \frac{(G^t)_{pq}^2 + G_{pq}^2}{(G^t)_{pq}} \tag{24}$$

From the common inequality $x \geq 1 + \log x$ with $x > 0$, we derive:

$$\begin{aligned} tr(GP^-G^T) &= \sum_{pqk} P_{qk}^- G_{pq} G_{pk} = \sum_{pqk} P_{qk}^- (G^t)_{pq} (G^t)_{pk} \frac{G_{pq} G_{pk}}{(G^t)_{pq} (G^t)_{pk}} \\ &\geq \sum_{pqk} P_{qk}^- (G^t)_{pq} (G^t)_{pk} \left(1 + \log \frac{G_{pq} G_{pk}}{(G^t)_{pq} (G^t)_{pk}}\right) \end{aligned} \tag{25}$$

$$2\mathrm{tr}(G^T Q^+) = 2 \sum_{pq} Q_{pq}^+ G_{pq} = 2 \sum_{pq} Q_{pq}^+ (G^t)_{pq} \frac{G_{pq}}{(G^t)_{pq}} \geq 2 \sum_{pq} Q_{pq}^+ (G^t)_{pq} \left(1 + \log \frac{G_{pq}}{(G^t)_{pq}}\right) \tag{26}$$

By combining inequalities (23), (24), (25) and (26), it is obvious that the function $H(G, G^t)$ given by (22) satisfies $F(G) \leq H(G, G^t)$ and $F(G) = H(G, G)$. Hence, we prove that $H(G, G^t)$ is an auxiliary function of $F(G)$.

Proof (Theorem 1): Given $H(G, G^t)$ defined by (22), by forcing the derivative of $H(G, G^t)$ with regard to G to zero:

$$\left(\frac{\mathrm{d}H(G, G^t)}{\mathrm{d}G}\right)_{pq} = \frac{\partial H(G, G^t)}{\partial G_{pq}} = 2 \frac{(G^t P^+)_{pq} G_{pq}}{(G^t)_{pq}} + 2 \frac{Q_{pq}^- G_{pq}}{(G^t)_{pq}} - 2 \frac{(G^t P^-)_{pq} (G^t)_{pq}}{G_{pq}} - 2 \frac{Q_{pq}^+ (G^t)_{pq}}{G_{pq}} = 0 \tag{27}$$

Rearranging (27), we obtain:

$$G_{pq} = (G^t)_{pq} \sqrt{\frac{(G^t P^-)_{pq} + Q_{pq}^+}{(G^t P^+)_{pq} + Q_{pq}^-}} \tag{28}$$

In addition, the Hessian matrix given by (29):

$$\Phi = \frac{\mathrm{d}^2 H(G, G^t)}{\mathrm{d}G^2} \tag{29}$$

is a diagonal matrix and the $i * j$-th entry of its diagonal is:

$$\Phi_{ij} = 2 \frac{(G^t P^+)_{ij} + Q_{ij}^-}{(G^t)_{ij}} + 2 \frac{\left[(G^t P^-)_{ij} + Q_{ij}^+\right] (G^t)_{ij}}{G_{ij}^2} \tag{30}$$

It is obvious that $\Phi_{ij} \geq 0$ and hence Φ is a positive semi-definite matrix. That is to say, $H(G, G^t)$ is a convex function with regard to G, and its global minimum is given by (28). We reformulate (28) into matrix form:

$$G^{t+1} = \arg\min_{G} H(G, G^t) = G^{t+1} = G^t \odot \sqrt{\frac{G^t P^- + Q^+}{G^t P^+ + Q^-}} \tag{31}$$

Then, according to **Lemma 2** and **Lemma 3**, the sequence $\{F(G^t)\}$ is a nonincreasing under the iterative rule (31). In other words, the value of objective function (13) decreases monotonically under the update rule (14).

1.3 Analysis of the hyperparameters of SFPEL-LPI

In this subsection, we give the interpretation for the exponent η of θ. In general, we often add a free hyperparameter as a constraint for the regularization term in the objective function. For example, in our objective function (32), we use μ and λ as regularization coefficients to balance the trade-off of each regularization terms.

$$\min_{G_i, R} \|R - Y\|_F^2 + \mu \sum_{i=1}^{n} \left\|X_i G_i^T - R\right\|_F^2 + \lambda \sum_{i=1}^{n} \|G_i\|_{1,2}^2 \tag{32}$$

Similarly, when introducing the proposed ensemble weighted graph Laplacian regularization term, we add a free parameter η as regularization coefficient. So we have:

$$\min_{\theta} \eta \sum_{i=1}^{m} \theta_i tr(R^T (D_i - W_i) R) \tag{33}$$

$$\text{s.t.} \sum_{i=1}^{m} \theta_i = 1$$

Then, we try to develop optimization rule to solve the problem (33). For simplicity, we denote $tr(R^T(D_i - W_i)R)$ as tr_i, and $tr_k = min(tr_1, tr_2, \cdots, tr_i, \cdots, tr_m)$. It is obvious that this problem is standard linear programming, and its solution is that $\theta_i = 1$ while $i = k$; $\theta_i = 0$ while $i \neq k$. Apparently, the solution fails to satisfy our demand. To ensure that all the graph Laplacian regularization terms contribute effectively for the maintaining of graph local structure, we rewrite the (33) by enforcing the hyperparameter η as the exponent of θ and transforming the standard linear programming into a nonlinear programming problem:

$$\sum_{i=1}^{m} \theta_i^{\eta} tr(R^T (D_i - W_i) R) \tag{34}$$

$$\text{s.t.} \sum_{i=1}^{m} \theta_i = 1$$

The objective function (34) not only remains the capacity and the role of η which balances the weight of the graph Laplacian regularization term in the model, but also makes it possible that we could obtain a satisfactory solution of θ. The details of the optimization rule of θ is fully presented in section 1.1.

Reference

1. Kuhn HW, Tucker AW: **Nonlinear Programming**. In: *Proceedings of the Second Berkeley Symposium on Mathematical Statistics and Probability: 1951 1951; Berkeley, Calif.*: University of California Press: 481-492.
2. Ding CHQ, Li T, Jordan MI: **Convex and Semi-Nonnegative Matrix Factorizations**. *IEEE transactions on pattern analysis and machine intelligence* 2010, **32**(1):45-55.
