# Supplementary material for: SFPEL-LPI: Sequence-based feature projection ensemble learning for predicting LncRNA-protein interactions
Source: PLoS Comput Biol. 2018 Dec 11;14(12):e1006616. doi: 10.1371/journal.pcbi.1006616 (PMC6331124; doi:10.1371/journal.pcbi.1006616)
Supplement: S1 Table — (DOCX) [file pcbi.1006616.s003.docx]

**S1 Table. Top 20 predictions of SFPEL-LPI and their ranks in predictions of benchmark methods**

| lncRNA | Protein | Confirmed? | SFPEL-LPI | LPBNI | KATZLGO | RWR | ETSLP-LPI | LPLNP |
| --- | --- | --- | --- | --- | --- | --- | --- | --- |
| NONHSAG003504 | 9606.ENSP00000258729 | Confirmed | 1 | 20 | 87 | 73 | 19 | 5 |
| NONHSAG103817 | 9606.ENSP00000258729 |  | 2 | 26 | 86 | 41 | 36 | 22 |
| NONHSAG005688 | 9606.ENSP00000258729 | Confirmed | 3 | 56 | 95 | 29 | 7 | 14 |
| NONHSAG017952 | 9606.ENSP00000258729 | Confirmed | 4 | 36 | 88 | 23 | 17 | 33 |
| NONHSAG036745 | 9606.ENSP00000258729 |  | 5 | 93 | 92 | 17 | 15 | 26 |
| NONHSAG043250 | 9606.ENSP00000258729 | Confirmed | 6 | 52 | 118 | 51 | 10 | 20 |
| NONHSAG033214 | 9606.ENSP00000258729 |  | 7 | 69 | 85 | 70 | 9 | 30 |
| NONHSAG050423 | 9606.ENSP00000258729 | Confirmed | 8 | 192 | 103 | 24 | 24 | 68 |
| NONHSAG004868 | 9606.ENSP00000290341 |  | 9 | 24 | 24 | 664 | 62 | 11 |
| NONHSAG099742 | 9606.ENSP00000258729 |  | 10 | 119 | 89 | 52 | 46 | 43 |
| NONHSAG044022 | 9606.ENSP00000258729 |  | 11 | 193 | 101 | 25 | 28 | 63 |
| NONHSAG021408 | 9606.ENSP00000258729 | Confirmed | 12 | 91 | 91 | 185 | 44 | 28 |
| NONHSAG053387 | 9606.ENSP00000258729 | Confirmed | 13 | 191 | 100 | 26 | 43 | 61 |
| NONHSAG016957 | 9606.ENSP00000258729 |  | 14 | 164 | 113 | 37 | 13 | 19 |
| NONHSAG027244 | 9606.ENSP00000258729 |  | 15 | 188 | 90 | 30 | 50 | 46 |
| NONHSAG047109 | 9606.ENSP00000258729 | Confirmed | 16 | 169 | 117 | 20 | 34 | 60 |
| NONHSAG053806 | 9606.ENSP00000258729 | Confirmed | 17 | 170 | 116 | 19 | 86 | 69 |
| NONHSAG038290 | 9606.ENSP00000290341 |  | 18 | 41 | 32 | 553 | 61 | 31 |
| NONHSAG041027 | 9606.ENSP00000290341 |  | 19 | 37 | 17 | 574 | 135 | 16 |
| NONHSAG025378 | 9606.ENSP00000258729 | Confirmed | 20 | 365 | 99 | 10 | 22 | 79 |
