# Supplementary material for: SFPEL-LPI: Sequence-based feature projection ensemble learning for predicting LncRNA-protein interactions
Source: PLoS Comput Biol. 2018 Dec 11;14(12):e1006616. doi: 10.1371/journal.pcbi.1006616 (PMC6331124; doi:10.1371/journal.pcbi.1006616)
Supplement: S2 Table — (DOCX) [file pcbi.1006616.s004.docx]

**S2 Table. Top 10 interacting proteins of LncRNA “NONHSAT041930” (OIP5-AS1) predicted by SFPEL-LPI**

| Index | Protein ID | Uniprot ID | Protein Name | Go Terms | Score |
| --- | --- | --- | --- | --- | --- |
| 1 | 9606.ENSP00000385269 | Q15717 | ELAV-like protein 1 | GO:0003676, GO:0045727, GO:0048255, GO:0005654, GO:0003725, GO:0017091, GO:0003729, GO:0003723, GO:0043488, GO:0003730, GO:1990904, GO:0005634, GO:0042803, GO:0016020, GO:0005515, GO:0005737, GO:0005829, GO:0000398, GO:0007275, GO:0060965, GO:0035925, GO:2000036, GO:0019901, GO:0070935 | 0.999 - Known Interaction |
| 2 | 9606.ENSP00000258729 | O00425 | Insulin-like growth factor 2 mRNA-binding protein 3 | GO:0003723, GO:0003676, GO:0009653, GO:0006417, GO:0006810, GO:0048027, GO:0005515, GO:0005737, GO:0051028, GO:0043488, GO:0005829, GO:0003730, GO:0042035, GO:0005634, GO:0017148, GO:0045182, GO:0006412 | 0.7223 |
| 3 | 9606.ENSP00000371634 | Q9Y6M1 | Insulin-like growth factor 2 mRNA-binding protein 2 | GO:0003729, GO:0003676, GO:0003723, GO:0009653, GO:0006417, GO:0005515, GO:0048027, GO:0005737, GO:0051028, GO:0043488, GO:0005829, GO:0003730, GO:0042035, GO:0005634, GO:0017148, GO:0045182, GO:0005856 | 0.6078 |
| 4 | 9606.ENSP00000290341 | Q9NZI8 | Insulin-like growth factor 2 mRNA-binding protein 1 | GO:0048471, GO:0030425, GO:0003676, GO:0048027, GO:0010494, GO:0030529, GO:0097150, GO:0005654, GO:0022013, GO:0003729, GO:0003723, GO:0006403, GO:0043488, GO:0043197, GO:0003730, GO:0030424, GO:0042995, GO:0005634, GO:0045182, GO:0030426, GO:0030175, GO:0006417, GO:0005515, GO:0005737, GO:0005829, GO:0042035, GO:0017148, GO:0070934, GO:0006810, GO:0030027, GO:0070937, GO:0051028, GO:0010610 | 0.5728 |
| 5 | 9606.ENSP00000254108 | P35637 | RNA-binding protein FUS | GO:0048471, GO:0030425, GO:0031489, GO:0003676, GO:0005844, GO:0003677, GO:0005654, GO:0043025, GO:0003723, GO:0043197, GO:0005634, GO:0005515, GO:0030331, GO:0005737, GO:0000398, GO:0071277, GO:0043204, GO:0035255, GO:0046966, GO:0046872, GO:0044327, GO:0046965, GO:0003713, GO:0006355, GO:0042802 | 0.5682 - Known Interaction |
| 6 | 9606.ENSP00000240185 | Q13148 | TAR DNA-binding protein 43 | GO:0003676, GO:0035061, GO:0003677, GO:0071765, GO:0005726, GO:0005654, GO:0003690, GO:0003723, GO:0016607, GO:0001933, GO:0042981, GO:0003730, GO:0010629, GO:0005634, GO:0005515, GO:0045944, GO:0034976, GO:0005737, GO:0006397, GO:0003700, GO:0032024, GO:0006351, GO:0008380, GO:0001205, GO:0006366, GO:0051726, GO:0043922, GO:0070935, GO:0006355, GO:0042802 | 0.3919 |
| 7 | 9606.ENSP00000220592 | Q9UKV8 | Protein argonaute-2 | GO:0030054, GO:0030425, GO:0035280, GO:0003676, GO:0008022, GO:0005844, GO:0035087, GO:0010501, GO:0090502, GO:1905618, GO:0003743, GO:0005654, GO:0003725, GO:0003729, GO:0000993, GO:0070551, GO:0003723, GO:0009791, GO:0090625, GO:0004519, GO:0010628, GO:0005845, GO:0070578, GO:0010586, GO:1990904, GO:0005634, GO:0006412, GO:0035197, GO:0035278, GO:1901165, GO:0045766, GO:0006417, GO:0035198, GO:0016020, GO:0005515, GO:0004518, GO:0045944, GO:0016442, GO:0005737, GO:0005829, GO:0007223, GO:0006413, GO:0060213, GO:0060964, GO:0003727, GO:0035196, GO:0000932, GO:0030422, GO:1900153, GO:0031047, GO:0006351, GO:0000340, GO:0016787, GO:0046872, GO:0001047, GO:0090624, GO:0004521, GO:0035279, GO:0031054, GO:0045947, GO:0035194, GO:0035068, GO:0098808, GO:0006355, GO:0070062 | 0.3402 |
| 8 | 9606.ENSP00000381031 |  |  |  | 0.31 |
| 9 | 9606.ENSP00000401371 | P31483 | Nucleolysin TIA-1 isoform p40 | GO:0017091, GO:0008143, GO:0003676, GO:0003723, GO:0006915, GO:0048024, GO:0097165, GO:0005515, GO:0042036, GO:1903608, GO:0010494, GO:1904037, GO:0005737, GO:0005829, GO:0005654, GO:0008543, GO:0017148, GO:0005634 | 0.2757 |
| 10 | 9606.ENSP00000349428 | P26599 | Polypyrimidine tract-binding protein 1 | GO:0075522, GO:0006397, GO:0003676, GO:0003723, GO:0008187, GO:0033119, GO:0000381, GO:0005515, GO:0008380, GO:0016020, GO:0005730, GO:0048025, GO:0051148, GO:0005654, GO:0000398, GO:0016070, GO:0036002, GO:0008543, GO:0005634, GO:0070062 | 0.247 |
