# Supplementary material for: SFPEL-LPI: Sequence-based feature projection ensemble learning for predicting LncRNA-protein interactions
Source: PLoS Comput Biol. 2018 Dec 11;14(12):e1006616. doi: 10.1371/journal.pcbi.1006616 (PMC6331124; doi:10.1371/journal.pcbi.1006616)
Supplement: S3 Table — (DOCX) [file pcbi.1006616.s005.docx]

**S3 Table. Top 20 interacting lncRNAs of protein “9606.ENSP00000240185” (TAR DNA-binding protein 43) predicted by SFPEL-LPI**

| Index | Transcript ID | Transcript Name | Gene ID | Gene Name | Score |
| --- | --- | --- | --- | --- | --- |
| 1 | NONHSAT137541 | XIST:3 | NONHSAG054780 | XIST | 1 - Known Interaction |
| 2 | NONHSAT021830 | SNHG1:4 | NONHSAG008552 | SNHG1 | 0.9784 - Known Interaction |
| 3 | NONHSAT135796 |  | NONHSAG053892 |  | 0.9387 - Known Interaction |
| 4 | NONHSAT041921 | OIP5-AS1:3 | NONHSAG016639 | OIP5-AS1 | 0.9298 - Known Interaction |
| 5 | NONHSAT027070 | lnc-H2AFJ-4:2 | NONHSAG010565 | lnc-H2AFJ-4 | 0.8993 - Known Interaction |
| 6 | NONHSAT104991 | lnc-PANK3-13:2 | NONHSAG042176 | lnc-PANK3-13 | 0.8924 - Known Interaction |
| 7 | NONHSAT104639 | lnc-SLC36A1-5:1 | NONHSAG041985 | lnc-SLC36A1-5 | 0.8813 - Known Interaction |
| 8 | NONHSAT002344 | lnc-SFPQ-2:1 | NONHSAG001012 | lnc-SFPQ-2 | 0.8555 - Known Interaction |
| 9 | NONHSAT011652 | lnc-CACNB2-1:1 | NONHSAG005343 | lnc-CACNB2-1 | 0.8316 |
| 10 | NONHSAT022115 | NEAT1:14 | NONHSAG008670 | NEAT1 | 0.8079 - Known Interaction |
| 11 | NONHSAT056046 | SNHG16:11 | NONHSAG073380 | SNHG16 | 0.7721 |
| 12 | NONHSAT054716 | lnc-LUC7L3-6:1 | NONHSAG022204 | lnc-LUC7L3-6 | 0.7572 |
| 13 | NONHSAT001511 | LINC01355:1 | NONHSAG000648 | LINC01355 | 0.7487 |
| 14 | NONHSAT001953 | SNHG3:2 | NONHSAG000835 | SNHG3 | 0.7456 |
| 15 | NONHSAT009703 | lnc-FAM177B-1:1 | NONHSAG004391 | lnc-FAM177B-1 | 0.7306 |
| 16 | NONHSAT114444 | lnc-GTF3C6-1:3 | NONHSAG044599 | lnc-GTF3C6-1 | 0.7241 |
| 17 | NONHSAT138142 |  | NONHSAG055086 |  | 0.7202 |
| 18 | NONHSAT134595 | lnc-RC3H2-1:1 | NONHSAG053377 | lnc-RC3H2-1 | 0.7139 |
| 19 | NONHSAT101154 | lnc-RPL37-1:2 | NONHSAG040258 | lnc-RPL37-1 | 0.7035 - Known Interaction |
| 20 | NONHSAT084827 | TUG1:9 | NONHSAG033691 | TUG1 | 0.6879 - Known Interaction |
